# Supplementary material for: Integrated psychosocial, economic strengthening, and clinical service-delivery to improve health and resilience of adolescents living with HIV and their caregivers: Findings from a prospective cohort study in Zambia
Source: PLoS One. 2021 Jan 22;16(1):e0243822. doi: 10.1371/journal.pone.0243822 (PMC7822390; doi:10.1371/journal.pone.0243822)
Supplement: S1 Table — (DOCX) [file pone.0243822.s001.docx]

| ***Construct*** | **ALHIV** | | **Caregivers** |
| --- | --- | --- | --- |
|  | 5-9 years | 10-17 years |  |
| Basic social support across four domains | 0.61 | 0.41 | n/a |
| Depressive symptoms | 0.68 | 0.67 | n/a |
| Stigma and mistreatment | 0.76 | 0.59 | 0.74 |
| Negative community attitudes towards PLHIV | n/a | 0.87 | 0.86 |

**S1 Table**. Measures of internal consistency (Cronbach’s alpha) for social protection and psychosocial wellbeing constructs derived from MEASURE Evaluation’s OVC Survey Toolkit for adolescents living with HIV (ALHIV) and their adult caregivers at baseline (*N* = 544).

*‘n/a’ indicates construct was not measured for specific age group or population.*
