# Supplementary material for: Integrated psychosocial, economic strengthening, and clinical service-delivery to improve health and resilience of adolescents living with HIV and their caregivers: Findings from a prospective cohort study in Zambia
Source: PLoS One. 2021 Jan 22;16(1):e0243822. doi: 10.1371/journal.pone.0243822 (PMC7822390; doi:10.1371/journal.pone.0243822)
Supplement: Attachment — Submitted filename: renamed_d8eb5.docx [file pone.0243822.s002.docx]

**COMMENTS – Manuscript PONE-D-20-22311**

Title: “Integrated psychosocial, economic strengthening, and clinical service-delivery to improve health and resilience of young people living with HIV and their caregivers: Findings from a prospective cohort study in Zambia”

**Important note: This review pertains only to ‘statistical aspects’ of the study and so** **‘****clinical aspects’ [like medical importance, relevance of the study,** **‘clinical significance and implication(s)’ of the whole study, etc.] are to be evaluated [should be assessed] separately/independently. Further please note that any ‘statistical review’ is generally done under the assumption that (such) study specific methodological [as well as execution] issues are perfectly taken care of by the investigator(s). This review is not an exception to that and so does not cover clinical aspects {however, seldom comments are made only if those issues are intimately / scientifically related & intermingle with ‘statistical aspects’ of the study}. Agreed that ‘statistical methods’ are used as just tools here, however, they are vital part of methodology [and so should be given due importance].**

**COMMENTS:** Basically study on young people living with HIV in country like Zambia is most welcome and appreciable. Moreover, this study is very well planned (with correct methodology, etc.), however, I have few doubts:

- According to statement made on page 11, [“At endline (July–September 2018), study enumerators returned to households sampled at baseline for a follow-up interview using similar data collection materials”] end line survey was conducted during July–September 2018, then why this delay in publication?
- Execution of such community trials is generally ‘critical’ [mostly because ‘no control’ on comparison group/sample] and has to be undertaken more carefully. It is desirable to discuss particular/specific steps taken [to ensure valid data / data quality, etc]. They are not discussed adequately, in my opinion.
- Footnote of table-2 gives classification/categorization of P-vales [a p < 0.05; b p < 0.01; c p < 0.001] but is not used {in fact indicating this is not necessary as actual P-values are reported}.
- Although data analyses done are correct but whether could have been performed by simple methods [for example, data of table-3 analysed by Poisson regression with generalized estimating equations (reference 29 quoted is also excellent) but could have done {why make all ‘binary’, few could be measured in scale} by Wilcoxon’s Signed Ranked test (not Wilcoxon rank-sum test which is incidentally same as well-known Mann-Whitney test) or for ‘binary data by McNemar’s test]? In my opinion, analyses should be as simple as possible. Statistical techniques used simply for better understanding of your observations only, I guess.

With the backdrop of ‘limitations’ described on page 27, best possible work is done which is indeed appreciable/acceptable.
