# Supplementary material for: Integrated psychosocial, economic strengthening, and clinical service-delivery to improve health and resilience of adolescents living with HIV and their caregivers: Findings from a prospective cohort study in Zambia
Source: PLoS One. 2021 Jan 22;16(1):e0243822. doi: 10.1371/journal.pone.0243822 (PMC7822390; doi:10.1371/journal.pone.0243822)
Supplement: Attachment — Submitted filename: ZAMFAM_DID_R&R.docx [file pone.0243822.s004.docx]

***Reviewer #1:***

**COMMENTS: Basically study on young people living with HIV in country like Zambia is most welcome, important and appreciable. Moreover, this study is very well planned (with correct methodology, etc.), however, I have few doubts:**

**- According to statement made on page 11, [“At endline (July–September 2018), study enumerators returned to households sampled at baseline for a follow-up interview using similar data collection materials”] end line survey was conducted during July–September 2018, then why this delay in publication?**

We thank the reviewer for this observation. Initially, the cohort study was scheduled to receive funding for two additional rounds of data collection (through 2020). The funding was unexpectedly stopped in 2019 (during preparation for Round 3 data collection), and the data were analyzed retrospectively shortly thereafter and prepared for this manuscript during 2019 and 2020.

**- Execution of such community trials is generally ‘critical’ [mostly because ‘no control’ on comparison group/sample] and has to be undertaken more carefully. It is desirable to discuss particular/specific steps taken [to ensure valid data / data quality, etc]. They are not discussed adequately, in my opinion.**

We thank the reviewer for this observation. We acknowledge in our Limitations (Pg. 27-28) that the statistical relationships observed in this study are associations, and we cannot infer causality due to the non-randomized, non-controlled design of our study. The strength of our inferences, nonetheless, is derived from other contextual elements of our study’s design, which we have added to our Methods. We have included some discussion around the absence of multi-component interventions like ZAMFAM in the comparison communities of Eastern Province, which we verified through extensive stakeholder engagements (Pg. 9). Additionally, selection of sites in Eastern Province was informed by proximity of comparison sites to districts in Central Province where ZAMFAM activities were ongoing: districts in Eastern Province were selected as study sites because of their lack of proximity to study sites in Central Province, which mitigated potential intervention spillover effects. We have clarified these important study design attributes in our Methods (Pg. 9).

**- Footnote of table-2 gives classification/categorization of P-vales [a p < 0.05; b p < 0.01; c p < 0.001] but is not used {in fact indicating this is not necessary as actual P-values are reported}.**

We thank the reviewer for noting this error. This, in fact, was an artifact from an earlier iteration of the table. We have removed the footnote from Table 2.

**- Although data analyses done are correct but whether could have been performed by simple methods [for example, data of table-3 analysed by Poisson regression with generalized estimating equations (reference 29 quoted is also excellent) but could have done {why make all ‘binary’, few could be measured in scale} by Wilcoxon’s Signed Ranked test (not Wilcoxon rank-sum test which is incidentally same as well-known Mann-Whitney test) or for ‘binary data by McNemar’s test]? In my opinion, analyses should be as simple as possible. Statistical techniques used simply for better understanding of your observations only, I guess.**

We thank the reviewer for this comment. We implemented a Poisson regression model with generalized estimating equations for the analyses presented only in Table 5 to determine whether the changes we observed across key study indicators were significantly larger among program beneficiaries compared to non-beneficiaries. This type of model allowed us to calculate a ‘difference in difference’ coefficient and standard error that compares changes over time between beneficiaries and non-beneficiaries. The analyses presented in Tables 3 and 4, by contrast, facilitate comparisons over time but only within each study group (beneficiaries or non-beneficiaries). The objective of this preliminary analyses was to describe significant changes within each subgroup over time and to identify variables that would ultimately be introduced into multivariable analysis. We have added additional justification for the regression modeling approach in the Data Analysis sub-section of the Methods (Pg. 14).

***Reviewer #2:***

**HIV care and treatment. Program implementers convene health staff and community health workers (CHWs), identifying strategies for scaling HIV testing as well as viral load screening (VLS) and CD4+ count monitoring for those living with HIV. CHWs are additionally paired with YPLHIV to monitor and support adherence to care and treatment. Comment: What kind of strategies for scaling HIV testing were implemented here? Were the strategies uniform throughout the households?**

We thank the reviewer for this question. Enhanced home-based counseling and testing by CHWs through index case testing and partner notification services were used to increase HIV testing in ZAMFAM beneficiary households. These strategies were uniform across beneficiary households, as CHWs were guided by program standard operating procedures and training for enhanced counseling. We have clarified this on Page 7.

**Parenting. Contracting with a local women’s religious organization, program implementers train community staff on parenting skills, including child abuse and gender-based violence.
Community staff subsequently mobilize caregivers of YPLHIV and hold meetings to discuss these issues. Comment: How did this religious organization reach out households of non-believers? can you explain how study mitigated bias or untoward effects of using such organization with uptake of this initiative?**

We thank the reviewer for this comment. Zambia is a predominantly Christian nation with a vibrant religious presence in civil society. Per the 2018 Zambia Demographic and Health Survey (DHS), over 95% of the Zambian population identifies as Christian (Catholic or Protestant). Numerous religiously affiliated organization in Zambia receive bilateral funding to implement health and development projects, like ZAMFAM. Through the ZAMFAM project architecture, the contracted faith-based organization identified ZAMFAM households through the same project roster to which community health workers had access. Because of the omnipresence of faith-based organizations in Zambia, we have no reason to suspect any reticence or refusal from ZAMFAM-beneficiary households to uptake parenting programs based on the organization’s religious orientation. We have also changed the terminology used to describe this contracted organization (i.e., faith-based organization) to clarify it is not affiliated with a specific religious parish or church (Pg. 7).

**Food security. Farming inputs – including maize seed, legumes, cassava, sweet potatoes,
chickens, and goats – are provided to OVC and YPLHIV households. Comment: How did intervention contribute towards results in Table 3 under Food and Nutrition?**

The intervention impact model we have added to the manuscript (Figure 1) explicitly outlines the relationships between each intervention component and key health and wellbeing indicators. Farming inputs are designed to provide agricultural and nutritional support to households experiencing food insecurity, either as a result of poor agricultural output (from prolonged and regular droughts) or financial insecurity that materialize as hunger.

**Household economic strengthening. Loans and savings schemes are introduced into Village
Action Groups, allowing for members to borrow money for investments (e.g., in businesses,
essential household items) or purchase agricultural inputs at lower interest rates.
Comment: Did you provide cash as part of intervention for study participants to invest? if yes, how did you evaluate the project to see if the intervention worked?**

Cash was funneled into the Village Action Groups for income-generating activities only in the urban areas of Southern and Central Province where DAPP is implementing ZAMFAM activities. Just over half of beneficiary households in this study sample (restricted to Central Province) had access to a Village Action Group where cash loans were availed as “pass on gifts” to other families participating in loans or savings schemes. Cash grants were not provided directly for participants to invest; rather, all cash participants received were expected to be re-invested into the Village Action Groups for other families to use and were treated as loans. The mechanics of these investment schemes have been the subject of numerous studies in sub-Saharan Africa and were, therefore, not explicitly studied here. We have clarified this in our Limitations (Pg. 28). Implementation-related outcomes of ZAMFAM interventions are current foci of interest in a complementary qualitative study in Lusaka and Copperbelt Provinces.

**Psychosocial support. YPLHIV are grouped with their primary caregiver and a neighbor to
support the child’s adherence to ART and engagement with HIV services. Counselors and CHWs additionally receive specialized training to enhance their counseling skills Comment: what do you mean by being paired up by a neighbor? How did you handle disclosure issues here?**

To clarify, counselors and CHWs linked caregivers of adolescents living with HIV to a volunteer from a neighboring household. This neighbor communicated with counselors and CHWs to support the caregiver in monitoring the adolescent’s medication adherence and HIV care appointments. For adolescents who had not transitioned to adult HIV care, disclosure to non-household members was supported by CHWs and counselors following consent from adolescent’s caregivers. Among adolescents that had transitioned to adult HIV care, consent for disclosure was obtained from the adolescent directly.

**Four matched districts in Eastern Province – Nyimba, Chipata, Petauke, and Lundazi – were selected as non-intervention, comparison sites based on HIV prevalence estimates for children under 15 from 2016-17 Health Management Information System records of new HIV infections in selected districts as well as the districts’ geographic positioning along main thoroughfares. Comment: Did you look at other factors such as socio economic status of matching districts? Was there any spilling effect geographically?**

Districts in Eastern Province were not matched based on other socio-economic factors. We have added discussion of this to the Limitations (Pg. 27). Because the districts in Central and Eastern Province are located far from each other, we neither anticipated nor observed any geographic spillover effects.

**Table 1. Operationalization of key Zambia Family (ZAMFAM) Project outcome variables Comment: What criteria did you use to categorize 'definition' into each domain? For instance how did Depressive symptoms get aggregated into a single domain? How did you know definition of certain outcome truly define it? did you measure the internal consistency?**

We thank the reviewer for these questions. Measures of depression, stigma, social support, and attitudes towards PLHIV were derived from a validated MEASURE Evaluation’s OVC Survey Toolkit. Spousal violence and comprehensive HIV knowledge constructs were derived from ICF International’s Demographic and Health Surveys (DHS), and standard definitions from the DHS for these latter two indicators were applied. We have clarified the sources from which these measures were derived in the Methods (Pg. 12). At the author’s request, we present measures of internal consistency (Cronbach’s alpha) for each of the aggregated measures for adolescents living with HIV, by age group (5-9 years, 10-17 years), and adult caregivers are included in the Supplemental Information (S1 Table).

**Table 3-YPLHIV
Comment: Is it possible to disaggregrate your findings by age group with interest to variations in the younger population particularly children and youth to see how the change in key health and socio-economic well-being indicators had taken place after the intervention? The idea is to see if this intervention truly reached the intended population**

We thank the reviewer for this suggestion. Key indicators which were measured only for specific groups of adolescents living with HIV are indicated in the table and disaggregated to that age group. We have added footnotes to the tables to indicate which variables were measured only among specific age groups. As adolescents aged 5-17 years were the intended target population, we have aggregated these results in the analysis for indicators that were measured across age groups (5-9 years and 10-17 years). We, however, defer to the Editor to recommend whether results should be disaggregated by age group in Table 3.

**General comment: Can this intervention be scaled up to your country? and other similar settings?**

We thank the reviewer for this important question. Because our study did not explicitly measure implementation-related outcomes (e.g., fidelity to process/structure, cost, coverage) that would inform scale-up efforts, we cannot make inferences from our analysis about whether the intervention can be brought to scale. As noted in the Methods (Pg. 6), the intervention is currently being implemented in numerous districts in four Zambian provinces (Central, Copperbelt, Lusaka, Southern). An ongoing qualitative study of the ZAMFAM project aims to document implementation-related processes and trajectories to better ascertain project scalability. Insights gleaned from existing engagement with project stakeholders and beneficiaries has demonstrated ZAMFAM interventions can be scaled with clear and consistent operational frameworks with multi-sectoral buy-in (i.e., collaborating with district-level line ministries and civil society organizations to identify and prioritize the most vulnerable households in a catchment area) and sustained financial support from donors. As these elements shape project scalability, this information will be the focus of future publications. We have included some of these consideration in our Conclusions (Pg. 28).

***Reviewer #3:***

**The authors in this article seek to evaluate the impact of an integrated HIV intervention on the health and wellbeing of adolescents living with HIV and their caregivers in Zambia. They use a prospective cohort study design, and compare outcomes among participants in the intervention versus non-intervention areas as well as assess changes in these outcomes a year later. The intervention evaluated is a PEPFAR funded programme (ZAMFAM) that targeted five areas (HIV care and treatment, parenting, food security, household economic strengthening, psycho-social support).

Overall, this is paper is well written. I commend the authors for going beyond narrow HIV treatment outcomes and examining the effects of broader indicators.

I approve for publication with minor revisions (detailed below)**


Introduction
**- Kindly update guidelines to reflect latest UNAIDS goal ( 95-95-95 ).**

We have updated these goals in the introduction (Pg. 4).

**- I would recommend highlighting why these multi-component intervention and evaluations assessing health and wellbeing outcomes are important in the context of the SDGs.**

We thank the reviewer for this suggestion. We have added the following in the Introduction to address the importance of the presented analysis in the context of the Sustainable Development Goals:

*“An improved understanding of the mechanisms through which multi-component interventions influence HIV-related morbidity and socio-structural vulnerabilities (e.g., food insecurity, educational retention) is needed to identify optimal and sustainable solutions to cross-cutting health and development challenges.”* (Pg. 5).

**- Could the author describe current HIV cascade outcomes among YPLHIV and in the discussion relate findings to this?**

We thank the reviewer for this suggestion. As recommended, we have added the following to the Introduction (Pg. 4): *“Adolescents also fall substantially behind older adults in the HIV care continuum: fewer than half (40.2%) of 15-24-year-olds living with HIV know their HIV status (adults 35-49 years: 71.3%), and fewer than three-fourths (73.8%) of adolescents on HIV treatment are virally suppressed (adults 35-49 years: 89.8%).”*

At the reviewer’s request, we have also expanded discussion of the HIV care continuum indicators

**- There is a word missing from this sentence … “as these compound adversities can destabilize access to and retention in HIV services”**

We thank the reviewer for noting this and have corrected the sentence for clarity (Pg. 5).

**- Perhaps consider more standard terminology for the age-range under investigation. See UNAIDS definition on YPLHIV (it is aged 15-24 years). A more appropriate definition that is aligned to the study age range is adolescents living with HIV (ALHIV).**

Per the reviewer’s recommendation, we have updated the terminology of the age-range under investigation, referred to herein as adolescents living with HIV (ALHIV). This has also been changed in the manuscript’s title and accompanying tables/figures.

Methods
**- The authors highlight the theory of change of the programme. It would be good to include a model/figure highlighting key hypothesised pathways.**

We thank the reviewer for this suggestion. We have added an intervention impact model that explicitly links the intervention components to hypothesized study outcomes (Figure 1).

**- Definitions and measures of wellbeing have been noted in the HIV literature. The authors should highlight how this construct was conceptualised in their study and why. In addition, the term resilience is used with regards to the caregiver and environment. It would be important to define this construct.**

We thank the reviewer for this comment. Wellbeing was a multi-dimensional construct that, in addition to physical and mental health outcomes, also included other proximal and distal determinants of health outcomes, including nutrition, social welfare, and protective assets (e.g., education, food security). We have provided a rationale for this definition in the Methods (Pg. 11):

“*In addition to addressing HIV-related outcomes, ZAMFAM’s core service package tackles key social and structural factors driving ALHIV’s. Indicators of wellbeing, therefore, measure the distal (indirect) mechanisms that ZAMFAM interventions act upon (e.g., social support, depression) to bolster health outcomes in ALHIV.”*

We have also provided an overarching definition for resilience in the context of the ZAMFAM interventions in the Methods (Pg. 6-7):

“*By providing households with the resources and skills to meet unrelenting financial and social challenges that can traditionally destabilize health-seeking behaviors and healthcare engagement, ZAMFAM interventions seek to foster resilience among ALHIV and caregivers, so they can continue prioritizing their health needs in the face of overlapping adversities.”*

**- Misuse of the comma: “Dovetailing existing, nationally scaled interventions for OVC and YPLHIV in Zambia…”**

We have removed the comma from this sentence.

**- The authors in some sections describe the intervention as being “multi-sectoral”. I would suggest expanding on this. Which sectors were involved and how was the intervention implemented?**

We thank the reviewer for this comment. Upon further inspection, the appropriate terminology to describe this intervention is actually “multi-component”, as only the health and civil society sectors were truly engaged. We have updated the terminology throughout the manuscript.

**- The authors indicate “Importantly, comparison sites in Eastern Province did not have similar or comparable interventions to ZAMFAM at the time of study enrollment”. However, it is unclear how this was assessed.**

We thank the reviewer for this comment. This was assessed through key informant interviews and planning meetings with provincial and district health officers in Eastern Province, who oversee all donor-funded HIV-related interventions implemented in the catchment areas. We have clarified this in our Methods (Pg. 8).

**- Were measures of depression and stigma based on validated measures? If not, the authors should indicate this /highlight this under limitations.**

As stated on Pg. 11, these measures were derived from MEASURE Evaluation’s validated OVC Survey Toolkit. We have also clarified this in a footnote to Table 1, where these constructs and measures are described.

**- Data collection- it is unclear who administered the baseline and follow-up questionnaire. Were these CHWs that ran the intervention or an external group of fieldstaff not involved in intervention delivery?**

We thank the reviewer for this question. To clarify, data collectors not involved with intervention delivery were hired and trained to administer surveys at baseline and endline. CHWs facilitated introduction of field staff to beneficiary households in ZAMFAM catchment areas but were not involved in data collection activities for this study.

**- The authors should describe the reason for this modelling approach.**

In response to a similar point raised by Reviewer #1, we have included additional language in the Data Analysis sub-section of the Methods (Pg. 13-14) that describes the rationale for the modeling approach:

*“Regression coefficients from a time-by-group interaction term were population-averaged estimates, pooling fixed and random effects from repeated clustered observations, quantifying differences in key outcomes between study groups over time.”*

Results
**- What % of beneficiaries and non-beneficiaries resided in urban vs. rural areas?**

Residence type was nearly evenly distributed in the sample, with slightly over half of beneficiaries and non-beneficiaries (54.8%) residing in urban areas. No significant differences (*p*=0.301) in residence type were observed when comparing beneficiaries to non-beneficiaries. Residence characteristics have been added to Table 1.

**- As mentioned above, it would be important to highlight what informed the selection of indicators for categories in Table 3 (e.g. social protection and psycho-social wellbeing). Stigma, depression and social support are regarded as more correlates of wellbeing.**

We thank the author for pursuing clarity here. We developed thematic buckets in which to situate health and wellbeing outcomes, inspired by ZAMFAM’s theory of change and the intervention impact model provided in Figure 1. We agree with the reviewer that the majority, if not all, factors presented in the table are correlates of wellbeing; however, we present them in a separate thematic bucket from other correlates of wellbeing (i.e., education, labor) because of the specific and unique intervention components that tackle each of these outcomes.

**- Table 4 does not include the variable on depressive symptoms- was this not probed in the caregiver questionnaire?**

Depressive symptoms were only measured for adolescents living with HIV, as the items used for measuring depressive symptoms were validated only for populations younger than 17.

Discussion
**- Could the authors describe why receipt of ZAMFAM services was associated with decreases in the % of YPLHIV engaging in income generating activities?**

We thank the reviewer for this suggestion. We hypothesize these reductions are attributed to increased financial security in the households receiving ZAMFAM services (i.e., farming inputs, loans and savings groups). We have weaved these interpretations into the paragraph in our Discussion outlining improvements in financial security indicators among ZAMFAM-beneficiary households (Pg. 25).

**- The literature indicates positive spillovers with regards to caregiver wellbeing. Could the authors explain why the intervention was associated with wellbeing benefits for the caregiver and not YPLHIV?**

We thank the reviewer for addressing this important point. We have addressed this point in our Conclusion but have moved it to the beginning of the Discussion to bring quicker attention to these dynamics (Pg. 24-25): *“As many ZAMFAM intervention components interface directly with caregivers (e.g., parenting skills, loans and savings groups), potential benefits may manifest more quickly among the most proximal program recipients (i.e., caregivers), taking more time for these effects to cascade down to indirect, distal beneficiaries, including ALHIV. Additional rounds of data collection may be needed to estimate the magnitude and significance of sustained intervention impacts on beneficiaries over time.”*

**- Furthermore, could the authors explain why the intervention was not associated with positive HIV treatment and care effects for YPLHIV?**

As alluded to in the Discussion (Pg. 26) and as illustrated in the results, HIV care and treatment outcomes were largely positive (>90%) among beneficiaries and non-beneficiaries across provinces. As a result, significant improvements in these outcomes could not be detected with the sample size available for this study. This could be an artifact of selection bias, and we have expanded discussion of these biases in the Limitations (Pg. 27).

**- Could authors explain further what other sources could have resulted in the declines in school retention among beneficiaries?**

Declines in school retention may have also been an artifact of aging in the cohort. Among beneficiaries enrolled in school, the likelihood of observing school attrition increases with age. In Zambia, school attrition among children is largest after completion of primary school, prior to secondary school. Because adolescents included in this study are in the age range where school attrition is most likely to occur, it is conceivable to imagine school retention would decrease over time as school-going adolescents in the cohort get older and progress in school. We offer additional reflection of these dynamics in the Discussion (Pg. 27).

**- To help unpack the above, could the authors also highlight information regarding intervention dose? What % of caregivers or YPLHIV attended sessions related to intervention activities.**

We thank the reviewer for this recommendation. As mentioned in response to Reviewer #1 above, the funding for this study was prematurely suspended. Subsequent data collection waves were planned to measure frequency and coverage of specific intervention components. As a result, these measures are regrettably not available for this manuscript. We have added this to our Limitations.

**- Limitations- it would be important to highlight the impact of selection bias on the direction of study findings. It is unclear what is meant by the term “differential sampling”. The authors should discuss the generalizability of study findings.**

We thank the reviewer for these comments. We have clarified in the Limitations that “differential sampling” refers to different sampling approaches for recruiting beneficiaries and non-beneficiaries. We have also expanded discussion in our Limitations (Pg. 28) of how the different sampling approaches in each province contribute to potential selection bias.

**- A statement on future research is needed- particularly with regards to other implementation science outcome measures (feasibility, acceptability, costs).**

We thank the reviewer for the suggestion and have added the following to our Conclusion (Pg. 28): *“Furthermore, additional research into service delivery-related outcomes (e.g., fidelity to process and structure, program acceptability and coverage, cost and feasibility) is needed to better understand how study findings are related to the processes and mechanics of ZAMFAM implementation.”*
